# A necessary condition for the guarantee of the superiorization method

**Published:** 2025-02-06

**Authors:** Kay Barshad, Yair Censor, Walaa Moursi, Tyler Weames, Henry Wolkowicz

**Affiliations:** 1 Department of Mathematics, University of Haifa, Mt. Carmel, Haifa 3498838, Israel; 2 Department of Combinatorics and Optimization, Faculty of Mathematics, University of Waterloo, Waterloo, Ontario, Canada N2L 3G1

**Keywords:** Feasibility-seeking, superiorization, bounded perturbations resilience, dynamic string-averaging, strict Fejér monotonicity, guarantee question of superiorization

## Abstract

We study a method that involves principally convex feasibility-seeking and makes secondary efforts of objective function value reduction. This is the well-known superiorization method (SM), where the iterates of an asymptotically convergent iterative feasibility-seeking algorithm are perturbed by objective function nonascent steps. We investigate the question under what conditions a sequence generated by an SM algorithm asymptotically converges to a feasible point whose objective function value is superior (meaning smaller or equal) to that of a feasible point reached by the corresponding unperturbed one (i.e., the exactly same feasibility-seeking algorithm that the SM algorithm employs.) This question is yet only partially answered in the literature. We present a condition under which an SM algorithm that uses negative gradient descent steps in its perturbations fails to yield such a superior outcome. The significance of the discovery of this “negative condition” is that it necessitates that the inverse of this condition will have to be assumed to hold in any future guarantee result for the SM. The condition is important for practitioners who use the SM because it is avoidable in experimental work with the SM, thus increasing the success rate of the method in real-world applications.

## 1 Introduction

The superiorization method (SM) interlaces objective function value reduction steps (called “perturbations”) into a feasibility-seeking algorithm (called the “basic algorithm”), creating a, so called, “superiorized version of the basic algorithm”. These steps cause the objective function to reach lower values locally, prior to performing the next feasibility-seeking iterations. A mathematical guarantee has not been found to date that the overall process of the superiorized version of the basic algorithm will not only retain its feasibility-seeking nature, but also accumulate and preserve globally the objective function reductions. For more information concerning the SM see, for example, [11], [12], [22], [25], [31] and [38].

Numerous works that are cited in [11] show that this global function reduction of the SM occurs in practice in many real-world applications. In addition to a partial answer to the guarantee of lower objective function values given in [19] with the aid of the concentration of measure principle, there is also the partial result of [22, Theorem 4.1] about strict Fejér monotonicity of sequences generated by an SM algorithm.

In the SM, any sequence generated by the superiorized version of a bounded perturbation resilient (see Definition 3.1 below) iterative feasibility-seeking algorithm converges to a feasible point. The “guarantee question of the SM” is to find conditions that assure that the objective function value at this point is smaller or equal to that of a point to which the SM algorithm would have converged if no perturbations were applied, everything else being equal.

In the preliminaries Section 3 we provide, for the reader’s convenience, a compact brief review about the SM and bounded perturbations resilience of algorithms and present the Dynamic String-Averaging projection (DSAP) feasibility-seeking algorithmic scheme of [22] that is used in this paper. Recent reviews on the topic appear in [28] and [12].

In spite of all the above positive statements, examples of cases where the SM fails have been constructed, see [2,38]. In this context, “fails” means that the guarantee question stated above did not hold. So, the quest for recognizing the properties of such situations in order to make statements on the guarantee problem of the SM continues.

Proving mathematically a guarantee of global objective function value reduction of the SM, compared to running its feasibility-seeking algorithm without perturbations, will probably require some additional assumptions on the feasible set. Such assumptions will apparently refer to the objective function, the parameters involved, or even to the set of permissible initialization points. We present in this note a “negative condition”, namely, a condition under which an SM algorithm that is based on the Dynamic String-Averaging Projection (DSAP) feasibility-seeking algorithmic scheme along with negative gradient descent steps in its perturbations, will fail to yield a superior outcome.

The significance of this negative condition is twofold. On one hand, it necessitates that a reverse statement that will nullify it will have to be assumed to hold in any potential future guarantee result for the SM. On the other hand, we show that when this condition does hold, then the SM algorithm can produce a superior result only if it converges to an optimal point. The latter statement is related to the alternative in Theorem 4.1(a) of [22], reproduced in Theorem 3.6 in Section 3 below.

Although this note discusses theory, we care to make a comment about computational aspects. In numerical examples one should present large-size problems because for small problems many algorithms “work” and performance improvements start to show when the problems are very large and quite sparse. We care to mention this because this is why the SM was envisioned in the first place. For example, in [10] it is shown that the advantage of an SM algorithm (called there “LinSup” for Linear Superiorization) for the data of a linear program, over an LP algorithm (Malab’s Simplex) increases as the problem sizes grow. Many papers referenced in the continuously updated bibliography page on the SM and perturbation resilience of algorithms [11] attest to the success of SM algorithms in a variety of real-world practical large-size problems.

The original motivation of the SM was, and still is, to handle situations in which reaching a feasible point in the nonempty intersection of finitely many sets, in a tractable manner, is the principal task. In such situations the SM provides a low cost perturbation that preserves the convergence of the perturbed feasibility-seeking algorithm to a feasible point while aiming to improve, aka superiorize, (reduce, not necessarily optimize) the value of an objective function. Methods to increase the effectiveness of the objective function reduction steps in SM algorithms, without invalidating the bounded perturbation resilience of the embedded feasibility-seeking algorithm, have recently been published (see, for instance, [2], [4], [19] and [25]). These include a randomization approach and an approach with restarts of the step-sizes.

Support for the reasoning of the SM may be borrowed from the American scientist and Noble-laureate Herbert Simon who was in favor of “satisficing” rather than “maximizing”. Satisficing is a decision-making strategy that aims for a satisfactory or adequate result, rather than the optimal solution. This is because aiming for the optimal solution may necessitate needless expenditure of time, energy and resources. The term “satisfice” was coined by Herbert Simon in 1956 [37], see also: https://en.wikipedia.org/wiki/Satisficing.

The paper is structured as follows. In Section 2 we list a few (out of many) works that used the SM in practice and in Section 3 we provide, for the reader’s convenience, a compact brief review about the SM and bounded perturbations resilience of algorithms. Recent reviews appear in [28] and [12]. In Section 3 we also summarize the Dynamic String-Averaging Projection (DSAP) feasibility-seeking algorithmic scheme of [22], i.e., the algorithm that is used in this paper. The negative condition and its consequences appear in Section 4.

## 2 Some applications that use the superiorization methodology

In [23], Guenter et al. consider the fully-discretized modeling of an image reconstruction from projections problem that leads to a huge and very sparse system of linear equations. Solving such systems, sometimes under limitations on the computing resources, remains a challenge. The authors aim not only at solving the linear system resulting from the modeling alone, but consider the constrained optimization problem of minimizing an objective function subject to the modeling constraints. To do so, they recognize two fundamental approaches: (i) superiorization, and (ii) regularization. Within these two methodological approaches they evaluate 21 algorithms over a collection of 18 different “phantoms” (i.e., test problems), presenting their experimental results in very informative ways.

In [9] Fink et al. study the nonconvex multi-group multicast beamforming problem with quality-of-service constraints and per-antenna power constraints. They formulate a convex relaxation of the problem as a semidefinite program in a real Hilbert space. This allows them to approximate a point in the feasible set by iteratively applying a bounded perturbation resilient fixed-point mapping. Inspired by the superiorization methodology, they use this mapping as a basic algorithm, and add in each iteration a small perturbation with the intent to reduce the objective value and the distance to nonconvex rank-constraint sets.

Pakkaranang et al. [4] construct a novel algorithm for solving non-smooth composite optimization problems. By using an inertial technique, they propose a modified proximal gradient algorithm with outer perturbations and obtain strong convergence results for finding a solution of a composite optimization problem. Based on bounded perturbation resilience, they present their algorithm with the superiorization method and apply it to image recovery problems. They provide numerical experiments that show the efficiency of the algorithm and compare it with previously known algorithms in signal recovery.

Especially interesting is the recent work of Ma et al. [1] who propose a novel decomposition framework for **derivative-free optimization (DFO) algorithms** that significantly extends the scope of current DFO solvers to larger-scale problems. They show that their proposed framework closely relates to the superiorization methodology.

Many more publications on practical applications of the SM can be found in [11]; these include [31–34,36], to mention but a few.

## 3 Preliminaries: superiorization and dynamic string-averaging

In this section we provide some background concerning the superiorization methodology and dynamic string-averaging. In Subsection 3.1 we discuss the superiorization methodology and bounded perturbation resilience. In Subsection 3.2 we recall the convergence properties of the Dynamic String-Averaging Projection (DSAP) feasibility-seeking algorithm and its superiorized version, presented, respectively, in [21] and [22].

### 3.1 The superiorization methodology

The superiorization methodology (SM) was born when the terms and notions “superiorization” and “perturbation resilience”, in the present context, first appeared in the 2009 paper [16]. This followed its 2007 forerunner by Butnariu et al. [5]. The ideas have some of their roots in the 2006 and 2008 papers of Butnariu et al. [6–8] where it was shown that if iterates of a nonexpansive operator converge for any initial point, then its inexact iterates with summable errors also converge. Since its inception in 2007, the SM has evolved and gained prominence. Recent publications on the SM devoted to either weak or strong superiorization, though without yet using these terms, are: [5,13–16, 20,21,24,26,30]. Many of these contain a detailed description of the SM, its motivation, and an up-to-date review of SM-related previous work.

The Webpage^1^ [11] is dedicated to superiorization and perturbation resilience of algorithms and contains a continuously updated bibliography on the subject. It is a source for the wealth of work done in this field to date, including two dedicated special issues of journals [18] and [27].

Our recent review [12] can serve as an introduction to the SM; also [29] and [28] are very helpful. Just to make the continued reading here more convenient for the reader we give below some of the fundamental notions of the SM.

Throughout the rest of the paper we consider a real Hilbert space X with the norm ‖⋅‖ and a real-valued, convex and continuous function ϕ:X→R. For a point z∈X, we denote by ∂ϕ(z) the subgradient set of ϕ at z. For a point x∈X and a nonempty, closed and convex subset S of X, we denote by d(x,S) the distance of x to S.

Bounded perturbation resilience is now a topic of interest in the current literature (see, for instance, [35]). We recall its definition.

#### Definition 3.1 ([15, Definition 1]).

**Bounded perturbation resilience (BPR)**. Let Γ⊆X be a given nonempty subset of X. An algorithmic operator 𝓐:X→X is said to be bounded perturbations resilient with respect to Γ if the following is true: If a sequence ${\left\{x^{k}\right\}}_{k=0}^{\infty }$, generated by the basic algorithm $x^{k+1}:=𝓐\left(x^{k}\right)$, for all k≥0, converges to a point in Γ for all $x^{0}\in X$, then any sequence ${\left\{y^{k}\right\}}_{k=0}^{\infty }$ of points X that is generated by the algorithm $y^{k+1}=𝓐\left(y^{k}+\beta_{k}v^{k}\right)$, for all k≥0, also converges to a point in Γ for all $y^{0}\in X$ provided that, for all k≥0, $\beta_{k}v^{k}$ are bounded perturbations, meaning that $\beta_{k}\geq 0$ for all k≥0 such that $\sum_{k=0}^{\infty }\beta_{k}<\infty$, and that the vector sequence ${\left\{v^{k}\right\}}_{k=0}^{\infty }$ is bounded.

A basic algorithm of the form $x^{k+1}:=𝓐\left(x^{k}\right)$, for all k≥0, is said to be bounded perturbations resilient with respect to Γ if its algorithmic operator 𝓐 is bounded perturbations resilient with respect to Γ.

Algorithm 3.2, presented here, is a superiorized version of the basic (feasibility-seeking) algorithm governed by 𝓐.

**Algorithm 3.2.** Superiorized version of the basic (feasibility-seeking) algorithm governed by 𝓐.

***(0) Initialization: Let*** N *be a natural number and let* $y^{0}\in X$ *be an arbitrary user-chosen vector*.
***(1) Iterative step:*** *Given a current iteration vector* $y^{k}$, *pick an* $N_{k}\in \left\{1,2,\ldots ,N\right\}$ *and start an inner loop of calculations as follows:*
***(1.1) Inner loop initialization:*** *Define* $y^{k,0}=y^{k}$.
***(1.2) Inner loop step:*** *Given* $y^{k,n}$, *as long as* $n<N_{k}$, *do as follows:*
***(1.2.1)*** *Pick a* $0<\beta_{k,n}\leq 1$ *in a way that guarantees that*
$\sum_{k=0}^{\infty }\sum_{n=0}^{N_{k}-1}\beta_{k,n}<\infty .$
***(1.2.2)*** *Pick an* $s^{k,n}\in \partial \phi \left(y^{k,n}\right)$ *and define direction vectors* $v^{k,n}$ *as follows:*
$v^{k,n}:=\{\begin{array}{lll} -\frac{s^{k,n}}{\left\|s^{k,n}\right\|}, & if & 0\notin \partial \phi \left(y^{k,n}\right)\text{,}\\ 0, & if & 0\in \partial \phi \left(y^{k,n}\right). \end{array}$
***(1.2.3)*** *Calculate the perturbed iterate*
$y^{k,n+1}:=y^{k,n}+\beta_{k,n}v^{k,n}$ (3.1)
*and if* $n+1<N_{k}$ *set* n←n+1 *and go to* ***(1.2)***, *otherwise go to* ***(1.3)***.
***(1.3)*** *Exit the inner loop with the vector* $y^{k,N_{k}}$
***(1.4)*** *Calculate*
$y^{k+1}:=𝓐\left(y^{k,N_{k}}\right)$ (3.2)
*set* k←k+1 *and go back to* ***(1)***.

### 3.2 The convergence properties of the DSAP algorithm and its superiorized version

The Dynamic String-Averaging Projection (DSAP) method of [21] constitutes a family of algorithmic operators that can play the role of 𝓐 in Algorithm 3.2. For each i=1,2,…,m, let $C_{i}$ be a nonempty, closed and convex subset of X and denote by $P_{i}:=P_{C_{i}}$ the metric projection onto the set $C_{i}$. An index vector is a vector $t=\left(t_{1},t_{2},\ldots ,t_{q}\right)$ such that $t_{s}\in \left\{1,2,\ldots ,m\right\}$ for all s=1,2,…,q, whose length is ℓ(t)=q. The composition of the individual projections onto the sets whose indices appear in the index vector t is $P[t]:=P_{t_{q}}\cdots P_{t_{2}}P_{t_{1}}$, called a string operator.

A finite set Ω of index vectors is called fit if for each i∈{1,2,…,m}, there exists a vector $t=\left(t_{1},t_{2},\ldots ,t_{q}\right)\in \text{Ω}$ such that $t_{s}=i$ for some s∈{1,2,…,q}. Denote by 𝓜 the collection of all pairs (Ω,w), where Ω is a finite fit set of index vectors and w:Ω→(0,∞) is such that $\sum_{t\in \text{Ω}}w(t)=1$.

For any (Ω,w)∈𝓜 define the convex combination of the end-points of all strings defined by members of Ω

$$P_{\text{Ω},w}(x):=\sum_{t\in \text{Ω}}w(t)P[t](x)$$

for each x∈X. Let Δ∈(0,1/m), and fix an arbitrary integer $\bar{q}\geq m$. Denote by ${𝓜}_{*}\equiv {𝓜}_{*}(\text{Δ},\bar{q})$ the set of all (Ω,w)∈𝓜 such that the lengths of the strings are bounded and the weights are all bounded away from zero, i.e.,

$${𝓜}_{*}:=\left\{(\text{Ω},w)\in 𝓜|\ell (t)\leq \bar{q}\;\text{and}\;w(t)\geq \text{Δ},\forall t\in \text{Ω}\right\}.$$

The convergence properties and bounded perturbation resilience of the DSAP method were analyzed in [21].

**Algorithm 3.3.** The DSAP method with variable strings and variable weights

***Initialization:*** *Select an arbitrary* $x^{0}\in X$,
***Iterative step:*** *Given a current iteration vector* $x^{k}$ *pick a pair* $\left({\text{Ω}}_{k},w_{k}\right)\in {𝓜}_{*}$ *and calculate the next iteration vector* $x^{k+1}$ *by*
$x^{k+1}:=P_{{\text{Ω}}_{k},w_{k}}\left(x^{k}\right).$

The strong convergences of Algorithm 3.3 and its superiorized version, as demonstrated in Theorems 3.5 and 3.6 below, are based on the following bounded regularity condition, see [3, Definition 5.1]. This condition is always satisfied in the case where the space X is finite dimensional (see [3, Proposition 5.4]).

#### Condition 3.4.

Let ${\left\{C_{i}\right\}}_{i=1}^{m}$ be a family of nonempty, closed and convex subsets of X with a nonempty intersection C. For each ε>0 and M>0, there exists δ>0 such that for each x∈X, we have the following implication

$$\{\begin{array}{c} \|x\|<M\\ d\left(x,C_{i}\right)<\delta \;\text{for}\;\text{all}\;i=1,2,\ldots ,m \end{array}\Rightarrow d(x,C)<\varepsilon .$$

#### Theorem 3.5

([21, Theorem 12]). *Assume that* ${\left\{C_{i}\right\}}_{i=1}^{m}$ *is a family of nonempty, closed and convex subsets of* X *with a nonempty intersection* C, *satisfying* Condition 3.4. *Let* ${\left\{\beta_{k}\right\}}_{k=0}^{\infty }$ *be a sequence of non-negative numbers such that* $\sum_{k=0}^{\infty }\beta_{k}<\infty$, *let* ${\left\{v^{k}\right\}}_{k=0}^{\infty }\subset X$ *be a norm-bounded sequence, let* ${\left\{\left({\text{Ω}}_{k},w_{k}\right)\right\}}_{k=0}^{\infty }\in {𝓜}_{*}$ *for all* k=0,1,… *Then any sequence* ${\left\{y^{k}\right\}}_{k=0}^{\infty }$, *generated by the iterative formula*

$$y^{k+1}:=P_{{\text{Ω}}_{k},w_{k}}\left(y^{k}+\beta_{k}v^{k}\right),$$

*converges in the norm of* X, *and its limit belongs to* C. *That is*, Algorithm 3.3 *converges to a point in* C *and its algorithmic operator is bounded perturbation resilient with respect to* C.

Using the algorithmic operator of the DSAP feasibility-seeking Algorithm 3.3 as the algorithmic operator 𝓐 in Algorithm 3.2, we recover the following main Theorem 4.1 of [22].

#### Theorem 3.6

([22, Theorem 4.1]). *Assume that* ${\left\{C_{i}\right\}}_{i=1}^{m}$ *is a family of nonempty, closed and convex subsets of* X *with a nonempty intersection* C, *satisfying* Condition 3.4. *Set* $C_{\min}:=\{x\in C|\phi (x)\leq \phi (y)$ *for all* y∈C. *Let* $C_{*}\subseteq C_{\min}$ *be a nonempty subset of* $C_{\min}$, *let* $r_{0}\in (0,1]$ *and* $\bar{L}\geq 1$ *be such that, for all* $x\in C_{*}$ *and all* y *such that* $\|x-y\|<r_{0}$,

$$\left|\phi (x)-\phi (y)\right|\leq \bar{L}\|x-y\|.$$

*Further, suppose that* ${\left\{\left({\text{Ω}}_{k},w_{k}\right)\right\}}_{k=0}^{\infty }\subset {𝓜}_{*}$. *Then any sequence* ${\left\{y^{k}\right\}}_{k=0}^{\infty }$, *generated by* Algorithm 3.2, *the superiorized version of the DSAP algorithm, converges in the norm of* X *to a* $y^{*}\in C$. *And exactly one of the following two alternatives holds:*

- $y^{*}\in C_{\min}$.
- $y^{*}\notin C_{\min}$ and there exist a natural number $k_{0}$ and a $c_{0}\in (0,1)$ such that for each $x\in C_{*}$ and for each integer $k\geq k_{0}$,
$${\left\|y^{k+1}-x\right\|}^{2}\leq {\left\|y^{k}-x\right\|}^{2}-c_{0}\sum_{n=1}^{N_{k}-1}\beta_{k,n}.$$

This shows that ${\left\{y^{k}\right\}}_{k=k_{0}}^{\infty }$ is strictly Fejér-monotone with respect to $C_{*}$, i.e., since $c_{0}\sum_{n=1}^{N_{k}-1}\beta_{k,n}>0$, we get that for every $x\in C_{*}$, the inequality ${\left\|y^{k+1}-x\right\|}^{2}<{\left\|y^{k}-x\right\|}^{2}$ holds for all $k\geq k_{0}$. The strict Fejér-monotonicity however does not guarantee convergence to a constrained minimum point. Rather, it only says that the so-created feasibility-seeking sequence ${\left\{y^{k}\right\}}_{k=0}^{\infty }$ has the additional property of getting strictly closer, without necessarily converging, to the points of a subset of the solution set of the constrained minimization problem.

Published experimental results repeatedly confirm that global reduction of the value of the objective function ϕ is indeed achieved, without losing the convergence toward feasibility, see [5,13–16,20,21,24,26,30]. In some of these cases the SM returns a lower value of the objective function ϕ than an exact minimization method with which it is compared, e.g., [17].

## 4 The “negative condition” on the superiorization method

We consider the Dynamic String-Averaging Projection (DSAP) feasibility-seeking algorithmic scheme of [22] and its superiorized version, as presented above in Subsection 3.2. Speaking specifically about [22, Algorithm 4.1], we know that under the assumptions of Theorem 3.6, exactly one of two things must happen, i.e., alternative (i) or (ii). This is a non-constructive theorem because it tells nothing about when each of the alternatives can occur.

If alternative (i) holding is the case, then it is correct to say that the sequence generated by the superiorized version of the bounded perturbation resilient feasibility-seeking Algorithm 3.3 converges to a feasible point that has objective function value smaller or equal to that of a point to which this algorithm would converge if no perturbations were applied. This would be true in this case because $y^{*}\in C_{\min}$ and the feasibility-seeking algorithm cannot do better.

The question remains for the case of alternative (ii). Namely, can we give conditions under which, if alternative (ii) holds, we will have that $\phi \left(y^{*}\right)⩽\phi \left(x^{*}\right)$, where $x^{*}$ is the limit of the same feasibility-seeking algorithm that is used in this SM algorithm when it is run without perturbations. This rerun without perturbations must have everything else equal, such as initialization point, relaxation parameters, order of constraints within the sweeps, etc.

The desire to distinguish between the alternatives (i) and (ii) of Theorem 3.6 leads us to the next lemma which gives a condition under which a limiting feasible point cannot belong to the solution set of the constrained minimization problem min {ϕ(x)∣x∈C}. This is the “negative condition” eluded to above, under which an SM algorithm that is based on the Dynamic String-Averaging Projection (DSAP) feasibility-seeking algorithmic scheme, which uses negative subgradient steps in its perturbations, will fail to yield a superior outcome.

### Lemma 4.1.

*Let* ${\left\{C_{i}\right\}}_{i=1}^{m}$ *be a family of nonempty, closed and convex subsets of* X *with a nonempty intersection* C. *Let* D⊂C *be a nonempty subset of* C, *and let* r≥1 *be a real number. Assume that* ${\left\{y^{k}\right\}}_{k=0}^{\infty }$ *is any sequence generated by* Algorithm 3.2, *with positive step-sizes* ${\left\{{\left\{\beta_{k,n}\right\}}_{n=0}^{N_{k}-1}\right\}}_{k=0}^{\infty }$, *direction vectors* ${\left\{{\left\{v^{k,n}\right\}}_{n=0}^{N_{k}-1}\right\}}_{k=0}^{\infty }$, *and with* $y^{0}\in X$ *an arbitrary initialization point. Suppose that the family* ${\left\{C_{i}\right\}}_{i=1}^{m}$ *satisfies* Condition 3.4. *Then*

- *If* $\hat{c}\in C$ *is a point such that*
(4.1) $$\|y^{0}-\hat{c}\|\leq (r-1)\sum_{n=0}^{N_{0}-1}\beta_{0,n},$$
  *then the sequence* ${\left\{y^{k}\right\}}_{k=0}^{\infty }$ *satisfies*
(4.2) $$\|y^{k}-\hat{c}\|\leq r\sum_{\ell =0}^{k-1}\sum_{n=0}^{N_{\ell }-1}\beta_{\ell ,n}$$
  for all k≥1.
- *If, additionally*,
(4.3) $$d(\hat{c},D)>r\sum_{\ell =0}^{\infty }\sum_{n=0}^{N_{\ell }-1}\beta_{\ell ,n}$$
  *holds, then the limit point* $y^{*}$ *of the sequence* ${\left\{y^{k}\right\}}_{k=0}^{\infty }$ *(which exists by* Theorem 3.6*) satisfies* $y^{*}\notin D$.

*Proof*. (i) The proof is by induction on k. For k=1, we use (3.1), (3.2), (4.1), the nonexpansivity of the operator $P_{{\text{Ω}}_{0},w_{0}}$ and the boundedness of the sequence ${\left\{v^{0,n}\right\}}_{n=0}^{\infty }$, to obtain (since $\hat{c}\in C$)

$$\left\|y^{k}-\hat{c}\|=\|y^{1}-\hat{c}\|=\|P_{{\text{Ω}}_{0},w_{0}}\left(y^{0,N_{0}}\right)-P_{{\text{Ω}}_{0},w_{0}}(\hat{c})\right\|\;\;\;\;\leq \|y^{0}+\sum_{n=0}^{N_{0}-1}\beta_{0,n}v^{0,n}-\hat{c}\|\leq \|y^{0}-\hat{c}\|+\|\sum_{n=0}^{N_{0}-1}\beta_{0,n}v^{0,n}\|\;\;\;\;\leq \|y^{0}-\hat{c}\|+\sum_{n=0}^{N_{0}-1}\beta_{0,n}\leq r\sum_{n=0}^{N_{0}-1}\beta_{0,n}=r\sum_{\ell =0}^{k-1}\sum_{n=0}^{N_{\ell }-1}\beta_{\ell ,n}.$$

Suppose that k>1 and make the inductive assumption that

(4.4) $$\|y^{k-1}-\hat{c}\|\leq r\sum_{\ell =0}^{k-2}\sum_{n=0}^{N_{\ell }-1}\beta_{\ell ,n}.$$

Since $\hat{c}\in C$ (recalling (3.2)), we have

(4.5) $$\|y^{k}-\hat{c}\|=\|P_{{\text{Ω}}_{k-1},w_{k-1}}\left(y^{k-1,N_{k-1}}\right)-P_{{\text{Ω}}_{k-1},w_{k-1}}(\hat{c})\|.$$

By (3.1),

(4.6) $$y^{k-1,N_{k-1}}=y^{k-1}+\sum_{n=0}^{N_{k-1}-1}\beta_{k-1,n}v^{k-1,n}.$$

Since the operator $P_{{\text{Ω}}_{k},w_{k}}$ is nonexpansive, we obtain from (4.5) and (4.6)

(4.7) $$\|y^{k}-\hat{c}\|\leq \|y^{k-1}+\sum_{n=0}^{N_{k-1}-1}\beta_{k-1,n}v^{k-1,n}-\hat{c}\|.$$

By the triangle inequality and the boundedness of the sequence ${\left\{v^{k-1,n}\right\}}_{n=0}^{\infty }$, combined with (4.7), we obtain

(4.8) $$\|y^{k}-\hat{c}\|\leq \|y^{k-1}-\hat{c}\|+\sum_{n=0}^{N_{k-1}-1}\beta_{k-1,n}.$$

The inductive assumption (4.4) and inequality (4.8), imply (since r≥1)

$$\left\|y^{k}-\hat{c}\right\|\leq r\sum_{\ell =0}^{k-2}\sum_{n=0}^{N_{\ell }-1}\beta_{\ell ,n}+r\sum_{n=0}^{N_{k-1}-1}\beta_{k-1,n}=r\sum_{\ell =0}^{k-1}\sum_{n=0}^{N_{\ell }-1}\beta_{\ell ,n}.$$

This completes the induction and shows that (i) indeed holds.

(ii) Assume to the contrary that $y^{*}\in D$. Taking the limit, as k→∞, on both sides of (4.2) yields

$$\|y^{*}-\hat{c}\|\leq r\sum_{\ell =0}^{\infty }\sum_{n=0}^{N_{\ell }-1}\beta_{\ell ,n},$$

which contradicts (4.3), thus proving that $y^{*}$ cannot be in D. ☐

*Remark* 4.2. Lemma 4.1 shows that if (4.2) and (4.3) hold, then alternative (i) of Theorem 3.6 cannot hold. The case r=1 is of theoretical interest only because then the initialization point $y^{0}$ is feasible, contrary to the prevailing situation in applications wherein the feasibility-seeking is initialized outside the feasible set C.

The next corollary provides an insight into a necessary choice of an initialization point of Algorithm 3.2 in order to establish its convergence to a point in D.

### Corollary 4.3.

*Under the assumptions of* Lemma 4.1, *assume that the sequence* ${\left\{y^{k}\right\}}_{k=0}^{\infty }$ *generated by* Algorithm 3.2 *with step-sizes* ${\left\{{\left\{\beta_{k,n}\right\}}_{n=0}^{N_{k}-1}\right\}}_{k=0}^{\infty }$ *converges to a point* $y^{*}\in D$ *and there exists a point* $\hat{c}\in C$ *such that*

(4.9) $$d(\hat{c},D)>\sum_{k=0}^{\infty }\sum_{n=0}^{N_{k}-1}\beta_{k,n}.$$

*Then the following inequality holds*

(4.10) $$\|y^{0}-\hat{c}\|\geq \left(d(\hat{c},D){\left(\sum_{k=0}^{\infty }\sum_{n=0}^{N_{k}-1}\beta_{k,n}\right)}^{-1}-1\right)\sum_{N_{0}-1}^{N_{0}-1}\beta_{0,n}.$$

*Proof*. Clearly,

$$d(\hat{c},D)>r\sum_{k=0}^{\infty }\sum_{n=0}^{N_{k}-1}\beta_{k,n},$$

for any real $1\leq r<d(\hat{c},D){\left(\sum_{k=0}^{\infty }\sum_{n=0}^{N_{k}-1}\beta_{k,n}\right)}^{-1}$. Since the limit point of the sequence generated by Algorithm 3.2 belongs to D, we must have, by Lemma 4.1, that for each such r,

$$\|y^{0}-\hat{c}\|>(r-1)\sum_{n=0}^{N_{0}-1}\beta_{0,n}.$$

Inequality (4.10) now follows by (4.9). ☐

*Remark* 4.4. Note that Lemma 4.1 and Corollary 4.3 are, in particular, true for $D=C_{\min}:=\left\{x\in C|\phi (x)\leq \phi (y)\;\text{for}\;\text{all}\;y\in C\right\}$.

Under the assumption (4.9), Corollary 4.3 provides a non-trivial necessary condition, namely, (4.10), for the convergence of any sequence generated by Algorithm 3.2 to a point in D. However, the aforesaid condition is not sufficient to this end, even if the assumption (4.9) holds. This observation is demonstrated in the following example.

**Example 4.5.** Let X:=ℝ, let ϕ:X→X be defined by $\phi (x):=x^{2}$ for each x∈X, and define C:=[0,10]. Clearly, C is a closed and convex subset of ℝ. Note that ϕ is 20-Lipschitz continuous on C, that is, $\left\|\phi (x)-\phi \left(x^{\prime }\right)\|\leq 20\|x-x^{\prime }\right\|$ for each x, $x^{\prime }\in C$. Set $D:=C_{\min}=\left\{0\right\}$, $\hat{c}:=8\in C$, and define the sequence of step-sizes ${\left\{\beta_{k,0}\right\}}_{k=0}^{\infty }$ of length 1 (that is, $N_{k}=1$ for each k=0,1…) by $\beta_{k,0}:=2^{-k}$ for each k=0,1… We then have

$$d(\hat{c},D)=8>2=\sum_{k=0}^{\infty }\beta_{k,0}=\sum_{k=0}^{\infty }\sum_{n=0}^{N_{k}-1}\beta_{k,n}.$$

Now choose any $y^{0}\geq 13$. Then we have

$$\sum_{n=0}^{N_{0}-1}\beta_{0,n}=\beta_{0,0}=1\;\text{and}\;\left(d(\hat{c},D){\left(\sum_{k=0}^{\infty }\sum_{n=0}^{N_{k}-1}\beta_{k,n}\right)}^{-1}-1\right)=3.$$

Therefore,

$$\left\|y^{0}-\hat{c}\right\|\geq 3=\left(d(\hat{c},D){\left(\sum_{k=0}^{\infty }\sum_{n=0}^{N_{k}-1}\beta_{k,n}\right)}^{-1}-1\right)\sum_{n=0}^{N_{0}-1}\beta_{0,n}.$$

However, the sequence ${\left\{y^{k}\right\}}_{k=0}^{\infty }$, generated by Algorithm 3.2, satisfies for each positive integer k,

$$y^{k}=10-\sum_{\ell =1}^{k-1}2^{-\ell }\underset{k\to \infty }{\to }9\notin D.$$

Similarly, if we choose in this example $C:=\left[0,3\cdot 2^{-1}\right]$, $\hat{c}:=1\in C$, and keep all other settings the same, then Condition (4.9) fails and Condition (4.10) trivially holds. But the sequence ${\left\{y^{k}\right\}}_{k=0}^{\infty }$, generated by Algorithm 3.2, satisfies for each positive integer k,

$$y^{k}=3\cdot 2^{-1}-\sum_{\ell =1}^{k-1}2^{-\ell }\underset{k\to \infty }{\to }2^{-1}\notin D.$$

Taking D in Corollary 4.3 to be a level-set of the function ϕ within C, the next corollary follows.

### Corollary 4.6.

*Under the assumptions of* Lemma 4.1, *pick any* $x^{*}\in C$ *together with* $y^{0}\in X$, *and define* $D:=\left\{c\in C|\phi (c)\leq \phi \left(x^{*}\right)\right\}$. *Choose positive step-sizes* ${\left\{{\left\{\beta_{k,n}\right\}}_{n=0}^{N_{k}-1}\right\}}_{k=0}^{\infty }$ *and* $\hat{c}\in C$ *such that*

(4.11) $$d(\hat{c},D)>\sum_{k=0}^{\infty }\sum_{n=0}^{N_{k}-1}\beta_{k,n}$$

*and*

(4.12) $$\|y^{0}-\hat{c}\|<\left(d(\hat{c},D){\left(\sum_{k=0}^{\infty }\sum_{n=0}^{N_{k}-1}\beta_{k,n}\right)}^{-1}-1\right)\sum_{n=0}^{N_{0}-1}\beta_{0,n}.$$

*If* $y^{*}$ *is the limit point of a sequence* ${\left\{y^{k}\right\}}_{k=0}^{\infty }$ *generated by* Algorithm 3.2, *then* $y^{*}\notin D$, *that is*, $\phi \left(y^{*}\right)>\phi \left(x^{*}\right)$.

## 5 Conclusion

In this paper we presented a condition under which a superiorization method (SM) that uses negative gradient descent steps in its perturbations fails to yield a superior outcome. This means that if $x^{*}$ is the limit point of the feasibility-seeking algorithm without perturbations, then under conditions (presented in Corollary 4.6), the corresponding superiorization algorithm fails to reach a feasible point with objective function value smaller or equal than that of $x^{*}$.

The guarantee question of the SM is, to date, only partially resolved, but an inverse of this “negative condition” will have to be included and assumed to hold in any future mathematical guarantee claim for the SM. The condition is important for practitioners who use the SM because it is avoidable in experimental work with the SM, thus increasing the success rate of the method in real-world applications. In future practical implementations of users of the SM it would be advisable to choose the initialization point far enough from the feasible set, so as to avoid the negative condition from occurring.

## Data Availability

Data sharing not applicable to this article as no data sets were generated or analyzed during the current study.

## Footnotes

1 http://math.haifa.ac.il/yair/bib-superiorization-censor.html#top, last updated on December 26, 2024 with 191 items.
